# The European Open Science Cloud as a common good
 Potentials and limitations of this endeavour

**DOI:** 10.12688/openreseurope.19170.1

**Published:** 2025-01-22

**Authors:** Anna Bertelli, Melania Acciai, Giorgio Rossi

**Affiliations:** 1 University of Milan Department of Physics Aldo Pontremoli, Milan, Lombardy, Italy

**Keywords:** European Open Science Cloud, FAIR Data, AI, Science Policies and Funding, Common Good, Inequality.

## Abstract

The European Open Science Cloud (EOSC) is envisioned as a transformative platform for advancing Open Science, aimed at benefiting a diverse array of stakeholders, including researchers, innovators, institutions, and the broader public. To fully harness EOSC’s potential as a common good, capable of delivering services to the research community such to potentially transform the way scientific production and communication is done, we address critical barriers that may actually restrict the equitable access and the optimal use of such services. In particular, we emphasize that key resources as required to access and exploit EOSC’s advanced FAIR-data services – such as data-processing algorithms – are, in fact, intrinsically limited and the access will be competitive. Governance and funding of EOSC present challenges associated with its effective openness in terms of accessibility to resources for its advanced exploitation.

## Introduction

The European Open Science Cloud (EOSC) is a pioneering initiative intended to enhance European research by facilitating open access to data, analytical tools, and advanced algorithms for data processing and analysis. By embracing the FAIR (Findable, Accessible, Interoperable, Reusable) principles ( Wilkinson *et al.*, 2016), EOSC aims to foster a high-quality research ecosystem that supports both Open Science practices and advanced data services, essential for driving collaborative, transparent, and reproducible research.

EOSC faces the challenge of meeting the high expectations of the research community and policymakers, who view it as a catalyst for transparent, unbiased research and a driver of innovation across diverse research domains. EOSC is expected to support both curiosity-driven and mission-oriented investigations, making high-quality data and analytical tools accessible across fields.

As an instrument to promote Open Science, a critical objective of EOSC is to prevent “any particular actor from locking research into a proprietary, consolidated environment, which would recreate, at a different level, the enclosures of knowledge that Open Science seeks to eliminate” ( Mounier, 2022, p. 3).

To ensure that EOSC can fulfil its vision of inclusive and equitable access to research resources, it is essential to structure it in a way that maximises societal benefit. This will require comprehensive assessments of both scientific and economic impact, as well as a sustainable funding model that encourages broad use while minimising access barriers.

In this paper, we analyse the current efforts to establish EOSC as a tool to accelerate the implementation of Open Science. The EOSC should uphold the idea of science as a global common good, effectively contributing to the universally available, accessible, and usable character of knowledge. However, access to EOSC’s data and services can be restricted, and the resources necessary to fully utilise its advanced services are finite and rivalrous. These issues are explored in detail and potential solutions to address these challenges, promoting an inclusive and effective use of EOSC are proposed.

## EOSC as a common good for science

The European Open Science Cloud (EOSC) ^1^ is an ambitious, long-term initiative launched in 2015 by the European Commission, aimed at developing a web of FAIR Data and Services to support science across Europe. This initiative is, therefore, the result of a deliberate political will, responding to the growing need within the scientific community to reflect on its practices and limitations, and to align science increasingly with the principles of Open Science. It is also meant to support social and economic innovation in accordance with the European Research Area (ERA) ^2^ policies and the Sustainable Development Goals (SDGs) ^3^.

The term Open Science (OS) has become increasingly common in conferences, universities, and policy discussions related to science. But the exact definition of Open Science remains vague and somewhat a “moving target”. Following Fecher and Friesike (2013), OS is an umbrella term that captures the emerging trend and need within the scientific community, as well as in critical literature on scientific practices, to align science more closely with the idealized image we intuitively associate with it. In other words, it encapsulates all advocacies for a science that is more open – specifically, more accessible, collaborative, interdisciplinary, transparent, and inclusive.

### Why Open Science is good for science

The emergence of OS can be attributed to various factors, all rooted in scientific practice. Broadly speaking, these factors can be categorized into pragmatic reasons and value-driven reasons, with significant interconnection between the two ^4^.

The first group of reasons emphasizes how the scientific enterprise as a whole can benefit from implementing practices of transparency, openness, and collaboration.

The second group illustrates how a more open science practice aligns more closely with the ideals and expectations of science as a democratic enterprise, with results that are equally accessible and usable by all.

The EOSC initiative has the potential to advance the scientific community on all the aforementioned fronts. That it is an initiative capable of increasing productivity, efficiency, and transparency within the scientific community is already evident, even at the current embryonic stage of network of FAIR data and data services, offering a clear entry point and a searchable catalog.

On the other hand, its potential to enhance the real and perceived trustworthiness of science, as well as the accessibility and democratic nature of scientific practice, is both highly likely and desirable, provided that certain potential challenges in the rapidly evolving technological setting are addressed effectively. That is desirable is clearly stated, as an example, in the EOSC Steering Board’s Policy Paper on “Advanced Digitalisation of Research”:

Research reproducibility and data usability will be enhanced if the advanced digitalization of data collection, validation, analysis, and simulation will be developed and become commons of the research community enforcing the Open Science principles and policies, creating a critical mass of Quality Assessed FAIR Data (QAFAIRD) and research-objects enabling reliable and secure Artificial Intelligence, Machine Learning and Virtual Research Environments ( European Commission, 2024, p. 2).

The risks associated with the implementation phase of EOSC, however, need adequate attention and analysis.

The construction of the EOSC is currently envisioned as a Federation of Nodes, initiated by the European Commission through the procurement and launch of the EU-Node ^5^ as the foundational cornerstone. The EOSC Federation is defined as a “system of systems”, where the federated components, or nodes, can either be purpose-built, like the EU-Node, or derived from existing research data systems, such as those developed and operated by Research Infrastructures, e-Infrastructures, and other research data service organisations of national or institutional character. The EOSC Federation does not yet have a formal constitution; instead, it is evolving through a “learn-by-doing” approach during the aggregation of nodes. This iterative process will lead to the development of a shared set of principles and practices, as outlined in a Handbook ^6^, which is intended to be a dynamic reference document.

It is at this early stage of development of the EOSC Federation that that the associated risks connected with inclusiveness, sovereignty, robustness, security, and trust need to be understood and addressed.

## EOSC as a common good for society at large

The EOSC is designed to serve as a powerful instrument for open collaboration, transparent data lifecycles, and support for social and economic innovation. In this sense, we could say that the EOSC can be seen as an initiative aimed at the benefit of society, thus characterising itself as a common good. The concept of the “common good” can be understood through a philosophical lens:

[…] the common good is best understood as part of an encompassing model for practical reasoning among the members of a political community. The model takes for granted that citizens stand in a “political” or “civic” relationship with one another and that this relationship requires them to create and maintain certain facilities on the grounds that these facilities serve certain common interests ( Hussain & Kohn, 2024).

This definition, however, is quite generic; moreover, some other times, the EOSC is defined instead as a “public good”. For instance, in the “Opinion paper on EOSC and commercial partners” by the EOSC Steering Board expert group, we read: “The focus of the EOSC is to become a public good. Key contributions towards construction and future operation of EOSC are expected from public research organizations and services also from the private sector” ( European Commission, 2022, p. 3).

The terms “common good” and “public good”, address different aspects. The common good encompasses resources that may not serve every individual's immediate interest but contribute to collective well-being – such as universities, arts, and cultural institutions. The resources that constitute the common good address a unique set of shared interests that are fundamental to all citizens – interests that form the core of civic engagement and mutual responsibility ( Hussain & Kohn, 2024).

The term “public good”, on the other hand, specifically tackles the economics perspective rather than the philosophical one. In everyday speech, public goods are understood as collective goods that are provided by the state. But in economics a public good is defined as something that is non-excludable – meaning it is difficult, if not impossible, to prevent anyone from accessing it, and non-rivalrous – one person's use of it does not diminish its availability to others ( Samuelson, 1954). According to the public choice school of economics, the state should provide public goods only when the benefits are non-excludable and the enjoyment of the goods is non-rivalrous ( Olson, 1965; Samuelson, 1954).

This conceptual framework is useful for highlighting one aspect: if EOSC is composed of both non-excludable and excludable parts, in order for it to be a tool for OS, it is important to adopt policies that limit potential excludability. The excludability in fact may emerge from the conditions of access to EOSC by the research community at large. We will analyse these aspects in a more detailed manner in paragraph 2.2.

From this characterization of EOSC as a common or public good, it is evident that issues of capacity, inclusion and equality need to be taken into account. This could be trivial, since the EOSC was explicitly designed to broaden access for under-resourced researchers and, in theory, to reduce disparities among those with different levels of access to resources. Yet, this assumption warrants close examination against the real-world conditions EOSC faces.

### Inclusion and equality in the Open Science framework

In the scientific landscape – much like in society at large – inequality and exclusion remain pervasive. The ability to conduct research is contingent on access to material resources and enabling conditions which vary significantly across countries, regions, gender, and thematic sectors.

According to DiPrete and Eirich (2006), academia is particularly susceptible to what they term “cumulative advantage”, defined as “a general mechanism for inequality across any temporal process (...) in which a favorable relative position becomes a resource that produces further relative gains” (p. 271). This concept refers to a process in which a favorable starting position leads to further advantages, perpetuating inequality. Unequal access to knowledge, infrastructure, and resources exemplifies this dynamic ( DiPrete & Eirich, 2006, p. 271).

OS policies must acknowledge and address these realities. Power imbalances and historical inequalities in knowledge production will not vanish simply because OS initiatives are implemented ( Albornoz *et al.*, 2020). As Ross-Hellauer and colleagues caution, “uncritical narratives of openness may fail to address structural barriers in knowledge production, thereby perpetuating the cumulative advantage of dominant groups and the knowledge they produce” ( Ross-Hellauer *et al*., 2022, p. 3).

EOSC must address issues of inclusion and equality, as these are core to the principles of the OS movement, which EOSC seeks to embody and advance. For instance, the definition given in the UNESCO Recommendations on Open Science (2021) pose inclusion in a prominent position:

Open science is defined as an inclusive construct that combines various movements and practices aiming to make multilingual scientific knowledge openly available, accessible and reusable for everyone, to increase scientific collaborations and sharing of information for the benefits of science and society, and to open the processes of scientific knowledge creation, evaluation and communication to societal actors beyond the traditional scientific community ( UNESCO, 2021, p. 7).

According to Sabina Leonelli, the most appropriate way to manage Open Science practices would be to prioritise inclusion ( Leonelli, 2023). If inclusion is not considered as the starting point of this endeavour, it would then be too late to reach it. Leonelli points out that in most OS policy documents, the ultimate goal of enhancing transparency and quality (often defined as reproducibility) is to create an inclusive and equitable research process (see for instance Burgelman *et al*., 2019; European Commission, 2016; European Commission, 2018b; National Academies, 2018; United Nations, 2019). However, this ideal is rarely implemented in practice.

If the disparities resulting from cumulative advantage are not adequately addressed before the full implementation of EOSC, the initiative itself may result in another vehicle for reinforcing cumulative advantage for those in more privileged positions. This, in turn, would widen the gap between researchers with abundant resources and those with fewer, contradicting EOSC’s purpose.

Below, we examine why and how EOSC could fail reducing inequalities if the issue of inclusion is not properly addressed. One key factor is that global standards can intensify discrimination ( Leonelli, 2024): researchers who lack the resources to participate in the development and governance of data infrastructure are disproportionately impacted by OS. However, rather than on researchers’ participation in decision-making processes, our focus centers on issues more closely related to their material starting conditions.

We analyse the different components of the EOSC in view of their accessibility and usability for all researchers and identifying potential barriers that may limit equitable access.

### EOSC and its components

From a structural point of view, the EOSC presents aspects that in the economic theory would be defined as excludable and non-excludable, rivalrous and non-rivalrous. From the combination of these variables, it is possible to identify three components of the EOSC.

***Non-rivalrous and excludable component: scientific knowledge.*** Scientific knowledge is often classified as both a common and a public good ( Becerril Garcia, 2024; Boulton, 2021; International Science Council, 2024), though it fits the economic definition of a public good only in theory ( Stiglitz, 1999). In practice, various obstacles – such as restricted access and paywalls – limit universal access and equitable benefits from scientific knowledge.

In the case of EOSC, we observe that access by one researcher may not diminish access for others, characterising it as non-rivalrous,

To understand the non-rivalrous side of the EOSC, it is useful to draw a parallel with the Research Infrastructure (RI) paradigm ^7^. Public investments in establishing and operating RIs create a shared resource for competitive researchers, enabling them to leverage unique facilities to generate data and knowledge more efficiently, with a higher return on investment than typical research environments. However, access to RIs is inherently limited; they are rivalrous because their finite capacity requires access to be regulated, often through peer-reviewed selection processes that prioritise high-impact projects.

In contrast, the EOSC is designed to support an unfiltered, unrestricted number of users – initially targeting the research and educational sectors but ultimately expanding access to innovation-driven organisations and broader society. This open-access approach necessitates scaling up infrastructure costs to accommodate growing demand, but it also promises medium- to long-term benefits by fostering unconventional discovery, innovation, and a vibrant scientific culture.

Yet, EOSC’s principle of “as open as possible” – which replicates an OS principle – is subject to excludable aspects, including barriers like access fees or restricted-access platforms, based on own rules of the participating nodes, which limit accessibility. Other examples include insufficient infrastructure for data analysis and and a lack of expertise, which can limit the broad impact on research. These barriers often stem from material conditions that the (mainly methodological) shift associated with OS marginally tackle. Moreover, the knowledge generated through scientific research is frequently privatised, further exacerbating inequalities ( Florio, 2024).

Given these considerations, EOSC is better described as a “club good” ( Buchanan, 1965). While some EOSC services may indeed be non-excludable, others restrict access to a subset of users. EOSC strives to embody the ideal of a common good, aiming to offer services accessible and beneficial to all. However, this remains an aspirational goal, and access to the resources and services within EOSC is not automatically open to all; various conditions and barriers can influence who is able to exploit its offerings. Therefore, policies about EOSC implementation need to effectively minimize its excludable component.

***Rivalrous and excludable aspects: the infrastructural level.*** At a deeper level, the infrastructures, machines, and tools essential for generating and interpreting data – despite being publicly funded and managed – function as private goods. This is because their ownership and most likely their use is typically restricted to a selected group of individuals or organizations that have access to the necessary resources, whether financial, technical, or institutional. As a result, while the infrastructure itself might be publicly supported, its practical availability is often exclusive. This exclusivity extends beyond the tools themselves to include the energy required to power the entire research ecosystem, and particularly to support data generation, storage, and processing.

Current computing and data storage infrastructures, which are critical to scientific research, are largely dependent on national investments, and their capacity is often constrained. As demand for computational resources increases, there is a growing concern that these infrastructures may not scale quickly enough to meet the needs. The rise of technologies like Artificial Intelligence (AI), especially Generative AI tools and Large Language Models (LLMs), is exacerbating this situation. These tools require massive computational power and memory, alongside specialized ancillary software, to function. As the need for high-performance computing (HPC) escalates, the inequality in access to these resources does it too, as only some institutions with substantial funding or access to state-of-the-art own infrastructure, can deploy or maintain these technologies at scale.

Furthermore, as these technologies evolve, the focus of interoperability within the research ecosystem is shifting from ensuring compatibility between data formats to ensuring “machine actionability”. This shift means that the emphasis is no longer just on whether data can be shared or used by different researchers but on whether it can be acted upon directly by machines.

In light of these considerations on the material resources required for research, it can be stated that EOSC services, which are designed as open platforms for sharing scientific data, find themselves in a paradoxical position. In fact, they exhibit characteristics of rivalrous and excludable goods – much like private goods – due to their reliance on proprietary, resource-intensive technologies. The limited availability of critical resources, such as computing power and storage capacity, means that only those with the necessary financial and technical resources will be able to fully benefit from EOSC services. This disparity risks undermining the core principles of openness, accessibility, and equality that EOSC wants to foster.

### A focus on Artificial Intelligence

Artificial intelligence as a tool for scientific research represents an area that demands careful and forward-thinking consideration, particularly as technological advancements continue to reshape the landscape of research infrastructure. As we move further, it is increasingly likely that machines will become the primary users of the EOSC, while their human owners or operators will assume the role of meta-users. This shift in the nature of EOSC usage introduces significant challenges, particularly in the realm of data querying, which will increasingly involve AI systems capable of making automated, complex requests, perhaps overseeding the FAIR paradigm.

This raises important questions about what constitutes a legitimate query for machines exploiting the EOSC and what does not. It will be essential to explore the criteria that define such legitimacy – especially when these queries are driven by AI algorithms rather than human intent. In particular, we must address how to prevent that queries that do not meet certain ethical, technical or scientific standards, or, in other words, that push the boundaries of what is legally permissible or technically/scientifically feasible trigger the EOSC. The use of deep learning algorithms within the context of EOSC, trained on data that adhere to FAIR principles, represents a significant opportunity for the scientific community and a major step towards the development of secure and reliable AI instruments. Because of this EOSC must develop robust strategies to protect its data against malicious intrusions or harmful use. Generative AI creates synthetic data that will integrate the EOSC, with predictable effects of both positive and negative sign. The increase of information exchange of EOSC has intrinsically the same nature as any other communication technology of the past (e.g. printing books) which contributed to the spread of good knowledge in case of scientific books, but also created the background for the most negative episodes of humanity in case of fake-news and books spreading fake-stories. 

One key element in addressing this issue will be the development of an expanded concept of metadata. The metadata primarily focuses on the methods and attributes of data collection and are mandatory for reproducibility and reuse by the specialists. However, as AI becomes more integrated into research workflows, it will be essential for metadata to also capture the potential uses of the data, the contexts in which the data might be applied, the impacts that may result from their use, and the correlations that might emerge in different scenarios. We could refer to this expanded form of metadata as “hyper-metadata” – a set of data descriptors that complement domain specific metadata by offering a more comprehensive view of how data could be utilized and the broader consequences of its application. In well confined cases AI can run on abundant raw data and obtain reliable results, as e.g. in pulsar discovery from astronomical underexploited archives. A massive use of AI on raw data would on the other hand escape the FAIR principles and makes it necessary to be regulated by hyper-metadata filters to understand the output of the algorithms.

## Funding and sustainability challenges for the EOSC

The absence or the excessive delay of the EOSC would have significant adverse effects for the European research landscape. Studies have underscored the high social and economic costs of lacking an ecosystem for open and FAIR data ( European Commission, 2018a; Stott, 2014) ^8^. However, there remains a concern that existing inequalities in research infrastructure and resources across thematic, institutional, regional, and national levels could widen as a result of the EOSC. Those with an initial advantage in research capacity may be better positioned in the competition for knowledge production once EOSC services are in operation.

Consider this analogy: street lighting is a public service funded through previous investments in the production and distribution of electricity, with installation, maintenance, and energy costs integrated into a general municipal or national budget. Street lighting offers widespread benefits, though some people benefit more than others; for example, those in remote areas may receive less light and therefore rely more on their own sources. In much the same way, users of the EOSC benefit unevenly depending on their material resources and infrastructure.

A related issue is the potential for a free-rider problem. Although a combination of high demand, reciprocity, and altruism might encourage stakeholders to invest in the EOSC, there remains a risk that some entities could gain considerable benefits from its data without contributing proportionally. This could lead to an imbalance, where those who invest – such as taxpayers through European and national funding and research institutions via in-kind contributions – shoulder the costs, while non-contributors gain disproportionate advantages.

To address these challenges, it is essential to establish a sustainable public funding model for the EOSC and the infrastructure required to harness its resources effectively. A European strategy for the EOSC should guarantee equitable access to digital and AI services for all researchers, regardless of their institution, nationality, or field of study.

### The need of a sustainable public funding model

The current EOSC model assumes that pre-existing national investments will fulfill its initial needs, with national networks expected to provide adequate storage and communication capacity in its early phase. However, the EOSC Federation’s reliance on self-funding nodes is unlikely to be sustainable in the medium to long term, as it risks exacerbating competition and exclusion among researchers.

Within the current model, national or EU funding is generally restricted to early-to-middle-stage innovation: this is not ideal to keep pace with the rapid changes in the landscape. According to the Financial Sustainability Task Force Progress Report mandated by the EOSC Association ( 2022), for EOSC to be sustained, the European Commission, Member States, Research Funding Organizations and Research Performing Organizations need to review their existing funding mechanisms and explore and experiment with new funding mechanisms to help sustain a growing and maturing research ecosystem for science and society.

Overall, the statement contained in the report on “Solutions for a sustainable EOSC”, written in 2020 – when the “Minimum Viable EOSC” (MVE), which is now under construction, was being imagined, remains true:

[…] there are many potential funding schemes and mechanisms that could fund the different components of the MVE but each comes with its own constraints and integrating them into a comprehensive funding plan will be a challenge requiring effort of an entrepreneurial nature to actively seek funding opportunities and secure them ( European Commission, 2020, p. 17).

For instance, there are barriers ^9^ connected to in-cash contributions that can be overcome through a mixture of in-cash and in-kind, as done by Research Infrastructures. The sustainability of the full potential of an operational EOSC appears to require a hybrid economic solution with a mix of general public funding for research projects and of specific investments to cope with the energy-costing rivalrous services.

### The rise in AI-related costs and related funding

As an important quota of HPC lined to AI and FAIR-data exploitation will develop, the cost of supercomputers, memories and networks and the related energy cost will become an explicit element of the cost of the EOSC. This inevitably leads to rivalrous and excludable services to the research community, likewise the large Research Infrastructures.

To mitigate these challenges, the public sector must develop targeted strategies based on a comprehensive analysis of the specific needs of diverse research communities across different contexts and fields, and a continuous cost monitoring and outlook. Achieving this vision will require significant capital investment and a well-coordinated operational budget among Member States, Associated Countries, and the European Commission, especially as EOSC services will be accessible to all, irrespective of their contribution.

Let us have a look to the Research Infrastructure funding paradigm, particularly the one used by the European Strategy Forum on Research Infrastructures (ESFRI) for RIs ^10^. Open, competitive access to RIs has greatly contributed to the success and accessibility of these investments; however, no standardized funding model has been universally adopted, and research inequality has not been fully addressed through open access alone. Funding models for RIs often include mechanisms such as juste retour (where benefits are proportional to each country’s investment), reserved quotas for national users, and limited access quotas for users from non-contributing institutions or countries ( ESFRI Long-Term Sustainability Working Group, 2017).

These models inherently give researchers from well-resourced institutions in RI-owner countries a competitive edge in securing access, particularly when it comes to leading projects as principal investigators. Researchers from lower- and middle-income countries often face additional barriers in this competitive landscape, with fewer opportunities to leverage these high-cost facilities. This disparity illustrates how open, competitive access does not overcome inequalities based on institutional and national resources, in spite of the fact that scientific merit of proposals from all provenances brings value to the RI investment.

With the increasing reliance on AI and the vast infrastructure required to support its development within EOSC, a hybrid funding approach may be necessary, balancing open access with strategic funding models to support equitable participation. A framework that combines investment returns with access quotas might help ensure that researchers from diverse economic backgrounds can benefit from and contribute to these digital resources. Careful planning is needed to prevent the unintended consequence of reinforcing existing research inequalities and to enable EOSC to support a genuinely inclusive research environment across the EU and beyond.

## Conclusions

The geography of the digital realm is rich with both possibilities and challenges. It offers the potential for unprecedented collaboration, with data that can be revisited and exploited infinitely, enabling continuous innovation. Yet precisely because this digital landscape lacks tangible boundaries, it can sometimes create hard to cross barriers. Digital tools have the power to unlock significant opportunities for growth, but they do not reduce intrinsically the disparities between people.

To ensure that EOSC achieves its intended impact and limits the risks of exclusion, a coordinated effort is essential across multiple governance levels. Careful planning of governance of EOSC including resources and regulatory frameworks is needed to foster inclusivity within the EOSC. This implies understanding the necessary capital investments and operational budgets required from Member States, Associated Countries, and the European Commission, as EOSC services will be accessible to all, regardless of individual investment levels. Additionally, establishing a sustainable public funding model will be critical for supporting the EOSC infrastructure, maximising resource utilisation, and approximating the beneficial concept of EOSC as a common good.

## Disclaimer

The views expressed in this article are those of the authors. Publication in Open Research Europe does not imply endorsement by the European Commission.

## Ethics and consent

Ethics and consent were not required.

## Data Availability

No data are associated with this article.
