# Supplementary material for: The European Open Science Cloud as a common good Potentials and limitations of this endeavour
Source: Open Res Eur. 2025 Apr 11;5:19. Originally published 2025 Jan 22. [Version 2] doi: 10.12688/openreseurope.19170.2 (PMC11824896; doi:10.12688/openreseurope.19170.2)
Supplement: Peer review history

# Reviewer report: Reviewer response for version 2

**DOI:** 10.21956/openreseurope.21517.r53069

**Published:** 2025-04-16

**Authors:** Rodrigo Vargas Honorato

**Affiliations:** 1 Utrecht University, Utrecht, The Netherlands

No further comments

Where applicable, are recommendations and next steps explained clearly for others to follow? (Please consider whether others in the research community would be able to implement guidelines or recommendations and/or constructively engage in the debate)

Yes

Does the article adequately reference differing views and opinions?

Yes

Are all factual statements correct, and are statements and arguments made adequately supported by citations?

Yes

Is the rationale for the Open Letter provided in sufficient detail? (Please consider whether existing challenges in the field are outlined clearly and whether the purpose of the letter is explained)

Yes

Is the Open Letter written in accessible language? (Please consider whether all subject-specific terms, concepts and abbreviations are explained)

Yes

Reviewer Expertise:

Open Science, EU Projects

I confirm that I have read this submission and believe that I have an appropriate level of expertise to confirm that it is of an acceptable scientific standard.

# Reviewer report: Reviewer response for version 1

**DOI:** 10.21956/openreseurope.20746.r50341

**Published:** 2025-02-22

**Authors:** Rodrigo Vargas Honorato

**Affiliations:** 1 Utrecht University, Utrecht, The Netherlands

Overview: The article evaluates the European Open Science Cloud (EOSC) as a key initiative for Open Science, emphasizing its role as a common good while identifying challenges in governance, accessibility, and funding. It brings as key points; The risk of cumulative advantage, benefiting well-funded institutions disproportionately; discusses the AI’s impact on computational resources and ethical concerns; details the financial challenges, including the feasibility of a hybrid funding model and further discusses the need for inclusive governance to ensure equitable access.

 Rationale for the Open Letter: The rationale is provided in sufficient detail.

 Engagement with Different Perspectives: The open letter adequately balances different views.

 Support for Factual Claims: The factual statements are strongly supported. 

 Accessibility of Language: The language is clear and accessible.

 Clarity of Recommendations: The recommendations for the next steps are clearly explained. 

 The open letter is a well-researched contribution to the Open Science debate with a strongly supported statements.

Where applicable, are recommendations and next steps explained clearly for others to follow? (Please consider whether others in the research community would be able to implement guidelines or recommendations and/or constructively engage in the debate)

Yes

Does the article adequately reference differing views and opinions?

Yes

Are all factual statements correct, and are statements and arguments made adequately supported by citations?

Yes

Is the rationale for the Open Letter provided in sufficient detail? (Please consider whether existing challenges in the field are outlined clearly and whether the purpose of the letter is explained)

Yes

Is the Open Letter written in accessible language? (Please consider whether all subject-specific terms, concepts and abbreviations are explained)

Yes

Reviewer Expertise:

Open Science, EU Projects

I confirm that I have read this submission and believe that I have an appropriate level of expertise to confirm that it is of an acceptable scientific standard.

# Reviewer report: Reviewer response for version 1

**DOI:** 10.21956/openreseurope.20746.r50358

**Published:** 2025-02-12

**Authors:** Kristijan Armeni

**Affiliations:** 1 Johns Hopkins University, Baltimore, Maryland, USA

The authors discuss the European Open Science Cloud (EOSC) initiative as a common/public good and the implications of the framework for EOSC's research equity. They emphasize that equity is a core goal behind reproducibility requirements in open science and that initiatives like EOSC might fail to deliver equitable outcomes if the implementation and funding schemes do not follow suit.

 The authors turn to the concepts of common and public good and their exclusive nature. Due to the documented phenomenon of cumulative advantage, even open research infrastructure risks aggravating digital inequity: those who have more resources, have competitive advantage and can leverage more open resources that those who have less.

 The crux of the argument pertains to infrastructure and funding models. The authors argue that by their nature, infrastructure and materials goods (that would comprise the EOSC) are excludable (those who use it, take up space for others) and cannot guarantee equitable outcomes if equitable policies and funding models are not put in place.

 I find this commentary to be timely, well argued for, and for the most part clearly written. I think it would be stronger if it included concrete example(s) of EOSC usage (if it exists) that could be used throughout the commentary to highlight the problems the authors are addressing and possibly policy solutions. In general, a greater focus and discussion on potential solutions, which are currently outlined towards the end, would make the commentary stronger. I indicate these and some minor points in my comments below.

 Comments

 1. Clarifications. "Federation of nodes" -- are these actual computational nodes or are these metaphorical components? Could Google contribute a Node? Meta its data centers? It would be helpful to illustrate perhaps with a short example. You do provide links to the EOSC documents, but a self-contained example, if available, would be valuable. Likewise for the term "Research Infrastructures" which has a specific meaning, but will remain opaque to non-policy experts.

 2. Adding references to work on energy and AI. Specifically, when discussing relationship between AI and energy demands, consider adding references to some of the important work on AI computing infrastructure and energy, these references are a good starting point:

- Strubell et al, 2020 [ref 3]
- Kaack et al, 2021 [ref 1]
- Luccioni et al, 2024: [ref 4]

3. Excludable infrastructure and fair usage. You state: " *The limited availability of critical resources, such as computing power and storage capacity, means that only those with the necessary financial and technical resources will be able to fully benefit from EOSC services. This disparity risks undermining the core principles of openness, accessibility, and equality that EOSC wants to foster."*

 --> In high-performance computing (HPC), a common procedure for fair allocation of computing resources to its users already having access takes into account any user's history of usage etc. (Kay & Louder, 1988. A fair share scheduler [ref 2]). The EOSC cannot make infrastructure goods non-excludable, but to run with a practical example, would your argument imply that EOSC at the infrastructure level must implement something akin to the fair share policy? Would that even be possible in the EOSC participation/funding schema?

 4. Discussion of hybrid funding schemes. " *A framework that combines investment returns with access quotas might help ensure that researchers from diverse [...]*". Funding schemes have the highest leverage point to ensure equity and this seems a good place to provide a practical example of this solution. If authors know of and can provide a reference where this concept (access quotas etc.) and its practical feasibility is discussed, it would make this argument stronger.

 5. Typo. "characterising it as non-rivalrous," --> missing either a sentence or a period.

Where applicable, are recommendations and next steps explained clearly for others to follow? (Please consider whether others in the research community would be able to implement guidelines or recommendations and/or constructively engage in the debate)

Partly

Does the article adequately reference differing views and opinions?

Yes

Are all factual statements correct, and are statements and arguments made adequately supported by citations?

Partly

Is the rationale for the Open Letter provided in sufficient detail? (Please consider whether existing challenges in the field are outlined clearly and whether the purpose of the letter is explained)

Yes

Is the Open Letter written in accessible language? (Please consider whether all subject-specific terms, concepts and abbreviations are explained)

Yes

Reviewer Expertise:

cognitive neuroscience, AI, open science, data sharing

I confirm that I have read this submission and believe that I have an appropriate level of expertise to confirm that it is of an acceptable scientific standard.

## References

[1] : Aligning artificial intelligence with climate change mitigation. *Nature Climate Change* .2022;12(6) : 10.1038/s41558-022-01377-7 518-527 10.1038/s41558-022-01377-7

[2] : A fair share scheduler. *Communications of the ACM* .1988;31(1) : 10.1145/35043.35047 44-55 10.1145/35043.35047

[3] : Energy and Policy Considerations for Deep Learning in NLP. *ACL Anthology* . 10.18653/v1/P19-1355 10.18653/v1/P19-1355

[4] : Power Hungry Processing: Watts Driving the Cost of AI Deployment?. *Association for Computing Machinery* . https://doi.org/10.1145/3630106.3658542 10.1145/3630106.3658542

# Reviewer report: Reviewer response for version 1

**DOI:** 10.21956/openreseurope.20746.r50345

**Published:** 2025-02-07

**Authors:** Sonia Stefanizzi

**Affiliations:** 1 Università di Milano Bicocca, Milan, Italy

This article addresses a crucial issue in the context of European open science, that is, the role of the European Open Science Cloud (EOSC) as a common good for research and society. The authors provide a detailed analysis of the potential and limitations of EOSC, emphasizing aspects of inclusiveness, sustainability, and accessibility. The article is an important contribution to the open science debate, offering interesting insights into the potential and limitations of EOSC. However, more attention to the practical details of the proposed solutions and a closer look at the ethical and social implications would improve the robustness of the analysis. In particular, it is suggested that the criteria of inclusiveness be developed more. Although the issue of inclusiveness is central, the article does not explore in depth how existing inequalities can be concretely mitigated in the context of EOSC. It is suggested to provide practical examples or case studies of already successfully implemented inclusiveness policies that could be adapted to the EOSC context.

 The article touches on the topic of AI in a promising way, but does not fully develop the ethical and technical implications of its use within EOSC. It would be useful to elaborate on how AI may affect data governance, accessibility, and potential discrimination, and to propose strategies to manage these issues. In addition, proposals to ensure sustainable funding of EOSC should be expanded. In this regard, it would be fruitful to include a more in-depth discussion of hybrid funding models (e.g., public and private contributions), specifying potential practical applications and barriers.

 Stakeholder involvement (e.g., researchers, institutions) is mentioned, but the process through which these groups could contribute to EOSC governance could be better clarified. It is suggested that concrete mechanisms be described for including stakeholders in the decision-making process, such as the use of public consultations or participatory platforms.

 Finally, the importance of EOSC for improving scientific communication and collaboration is mentioned in the article but not explored in depth. It would be useful to discuss how EOSC can foster the dissemination of knowledge.

Where applicable, are recommendations and next steps explained clearly for others to follow? (Please consider whether others in the research community would be able to implement guidelines or recommendations and/or constructively engage in the debate)

Yes

Does the article adequately reference differing views and opinions?

Yes

Are all factual statements correct, and are statements and arguments made adequately supported by citations?

Yes

Is the rationale for the Open Letter provided in sufficient detail? (Please consider whether existing challenges in the field are outlined clearly and whether the purpose of the letter is explained)

Yes

Is the Open Letter written in accessible language? (Please consider whether all subject-specific terms, concepts and abbreviations are explained)

Yes

Reviewer Expertise:

Research infrastructures, open data, open government data, open science

I confirm that I have read this submission and believe that I have an appropriate level of expertise to confirm that it is of an acceptable scientific standard.

# Response

**Published:** 2025-02-11

**Authors:** Anna Bertelli

We would like to thank Prof. Stefanizzi for her overall apprecitiation of our work and for the stimulus to further explore the potential criticalities of EOSC.

 Our article highlights key aspects of EOSC that should be part of the public discourse during this crucial phase of the EOSC Federation's initial implementation, as well as decisions on funding strategy within the Framework Programme for research.

 Among the suggestions put forward by Prof. Stefanizzi we definitely strive to delve deeper into the criteria of inclusivity, the overall impact of AI, including in data management, in future works.

 We will not forget to include concrete examples and case studies, as suggested.
